# The Effect of Optic Disc Center Displacement on Retinal Nerve Fiber Layer Measurement Determined by Spectral Domain Optical Coherence Tomography

**DOI:** 10.1371/journal.pone.0165538

**Published:** 2016-10-26

**Authors:** Joong Won Shin, Yong Un Shin, Ki Bang Uhm, Kyung Rim Sung, Min Ho Kang, Hee Yoon Cho, Mincheol Seong

**Affiliations:** 1 Department of Ophthalmology, College of Medicine, Hanyang University, Seoul, Korea; 2 Department of Ophthalmology, Asan Medical Center, College of Medicine, University of Ulsan, Seoul, Korea; 3 Department of Ophthalmology, Hanyang University Guri Hospital, College of Medicine, Hanyang University, Guri, Korea; The University of Melbourne, AUSTRALIA

## Abstract

**Purpose:**

To investigate the effect of optic disc center displacement on retinal nerve fiber layer (RNFL) measurement determined by spectral domain optical coherence tomography (SD-OCT).

**Methods:**

The optic disc center was manipulated at 1-pixel intervals in horizontal, vertical, and diagonal directions. According to the manipulated optic disc center location, the RNFL thickness data were resampled: (1) at a 3.46-mm diameter circle; and (2) between a 2.5-mm diameter circle and 5.4-mm square. Error was calculated between the original and resampled RNFL measurements. The tolerable error threshold of the optic disc center displacement was determined by considering test-retest variability of SD-OCT. The unreliable zone was defined as an area with 10% or more variability.

**Results:**

The maximum tolerable error thresholds of optic disc center displacement on the RNFL thickness map were distributed from 0.042 to 0.09 mm in 8 directions. The threshold shape was vertically elongated. Clinically important unreliable zones were located: (1) at superior and inferior region in the vertical displacement; (2) at inferotemporal region in the horizontal displacement, and (3) at superotemporal or inferotemporal region in the diagonal displacement. The unreliable zone pattern and threshold limit varied according to the direction of optic disc displacement.

**Conclusions:**

Optic disc center displacement had a considerable impact on whole RNFL thickness measurements. Understanding the effect of optic disc center displacement could contribute to reliable RNFL measurements.

## Introduction

Accurate measurement of retinal nerve fiber layer (RNFL) thickness is an important component in the diagnosis and management of glaucoma. Due to the influence of eye motion and manually controlled circle placement, it is difficult to ensure consistent scan location in time-domain optical coherence tomography (TD-OCT). Accurate registration of circle scan is essential for measurement reproducibility and longitudinal examination [1]. Spectral-domain OCT (SD-OCT) automatically delineates the optic disc margin and calculates the center of the optic disc. Therefore, it consistently measures circumpapillary RNFL thickness along a 3.46-mm-diameter circle from the optic disc center. Due to these features, SD-OCT shows improved reproducibility of RNFL measurement compared to TD-OCT [2, 3].

However, automatic detection technique of optic disc in SD-OCT is not perfect. In previous studies using SD-OCT, the prevalence of optic disc margin detection errors was reported to range from 0.5 to 17.6% [4–6]. Because the optic disc center is calculated using the optic disc margin, error may also be incorporated in the optic disc center location. Potential displacement of the optic disc center may affect every measurement in the 200 x 200 scan points of a RNFL thickness map, as well as in a 3.46-mm-diameter circumpapillary RNFL measurement.

The purpose of this study is to investigate the effect of optic disc center displacement on RNFL measurement. Cirrus HD-OCT (Carl Zeiss Meditec, Dublin, CA) provides coordinate information of the optic disc center location in printout results. By shifting these coordinates, we simulated optic disc center displacement in the RNFL thickness map without requiring repeat scanning. Additionally, we present a strategy for how to deal with optic disc center displacement in clinical practice.

## Methods

### Participants

This prospective cross-sectional study was performed on healthy subjects who had visited the glaucoma clinic of the Hanyang University Medical Center from January 2015 to October 2015. The study was approved by the institutional review board of Hanyang University Medical Center and adhered to the tenets of the Declaration of Helsinki. Written informed consent was obtained from all subjects prior to participation.

All subjects underwent a comprehensive ophthalmic examination, including a visual acuity test, applanation tonometry, anterior segment examination, refraction, optic disc photography, standard automated perimetry (Humphrey Field analyzer with SITA standard 30–2 test; Carl Zeiss Meditec), and RNFL imaging with a SD-OCT (Cirrus HD-OCT; Carl Zeiss Meditec).

Inclusion criteria were as follows: best-corrected visual acuity of ≥20/40; normal visual field; normal anterior segment on slit-lamp examination; normal appearing optic nerve head (ONH); and no history of intraocular pressure >21 mmHg. Normal visual field was defined by a glaucoma hemifield test (GHT) within normal limits. Visual field tests were considered reliable based on fixation losses <20%, false-positive <15%, and false-negative <15%. Eyes with high myopic or hyperopic refractive errors of less than –6.0 diopters (D) or greater than +3.0 D were excluded from this study. Eyes with any ophthalmic or neurologic disease known to affect RNFL thickness or visual function were also excluded. If both eyes were eligible, one eye was randomly selected for the study.

### Retinal Nerve Fiber Layer Imaging

An Optic Disc Cube scan on the 6 x 6 mm^2^ optic disc area was obtained using a Cirrus HD-OCT. The RNFL thicknesses at 200 x 200 pixels were measured and a RNFL thickness map was generated. A built-in algorithm automatically detected the optic disc margin and calculated the geometric center of the optic disc margin. The optic disc center was displayed as the degree of movement from the center of 6 x 6 mm^2^ optic disc area in printout results (e.g. “Disc Center [-0.02, 0.04] mm”). Then, a circle 3.46 mm in diameter was positioned around the optic disc center, and circumpapillary RNFL thickness was calculated. Eyes with initial optic disc center position of >0.3 or <-0.3 mm were excluded. All images had signal strength of at least 7. Images with motion artifacts were rescanned at the same visit.

### Optic disc center displacement between optic disc photography and optical coherence tomography

**Fig 1:**
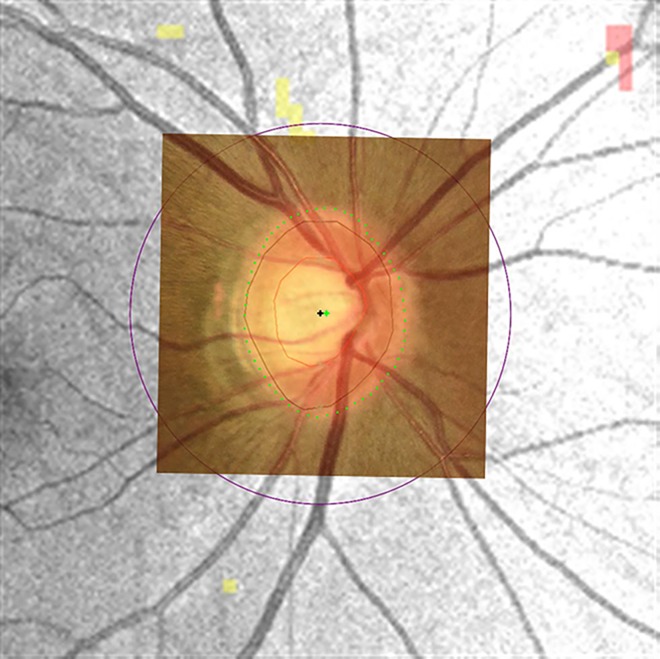
Optic disc center displacement between optic disc photography and optical coherence tomography (OCT). The infrared image of deviation map displayed the optic disc margin (black line) and center (black cross) information as well as abnormally deviated area from normative database. It was overlaid and registered with optic disc photograph according to the retinal blood vessels. Clinically visible optic disc margin (green dots) was segmented in optic disc photograph and its center (green cross) was calculated by averaging the coordinates of margin. In this case, clinically visible optic disc center was nasally displaced 1 pixel from the center of OCT image.

To compare the same region, the optic disc photograph and infrared image of OCT were registered by a control point selection tool according to the retinal blood vessels using MATLAB R2012a (The MathWorks Inc., Natick, MA). Clinically visible optic disc margin was segmented in optic disc photograph by one observer (J.W.S.) and reviewed with two observers (M.S. and K.B.U.). The optic disc center of optic disc photograph was calculated by averaging the coordinates of the optic disc margin segmentation. The optic disc margin from OCT built-in algorithm was compared with clinical optic disc margin from optic disc photograph by two observers (J.W.S and M.S). Obvious discrepancy between two disc margins was excluded. The difference of centration between optic disc photograph and OCT image was evaluated. Fig 1 showed the optic disc center displacement between optic disc photography and optical coherence tomography.

### Optic Disc Center Displacement and Retinal Nerve Fiber Layer Measurements

**Fig 2:**
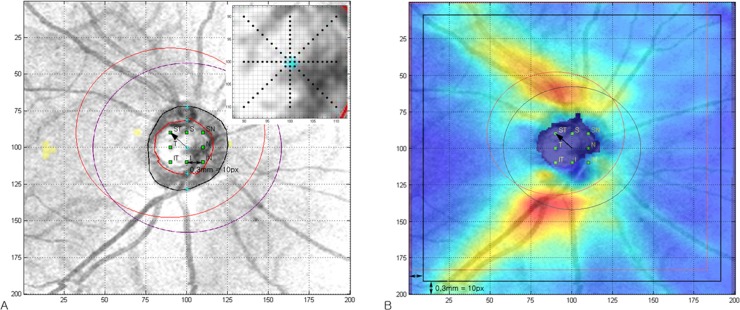
Optic disc center displacement and data sampling positional variation. (A) Cirrus HD-OCT’s built-in algorithm automatically calculated the optic disc center (central light-blue dot) and 3.46-mm circumpapillary scan location (purple circle). The optic disc center displacement (arrow, 0.42 mm superotemporal movement) caused a sampling position change (outer red circle) in circumpapillary RNFL thickness. In this study, we manipulated the optic disc center (green and black dots) from 0 to 0.3 mm at 0.03-mm intervals in horizontal (temporal and nasal) and vertical (superior and inferior) directions and 0 to 0.42 mm at 0.042-mm intervals in diagonal (superotemporal, superonasal, inferonasal, and inferotemporal) directions. This 0.03-mm (equal to 1 pixel) or 0.042-mm (equal to 1 diagonal pixel) interval was the minimum evaluable scale in Cirrus HD-OCT. (B) In RNFL thickness map, RNFL thickness data were resampled between a 2.5-mm diameter circle and a 5.4-mm square. According to optic disc center displacement, the original scan area (black circle and square) was adjusted to a new scan area (red circle and square). OCT = optical coherence tomography; RNFL = retinal nerve fiber layer; T = temporal; ST = superotemporal; S = superior; SN = superonasal; N = nasal; IN = inferonasal; I = inferior; IT = inferotemporal; px = pixel

RNFL thickness measurements were extracted from 200 x 200 pixels of the RNFL thickness map by a Cirrus HD-OCT Research Browser (Carl Zeiss Meditec). The sampling position of circumpillary RNFL measurement (Fig 2A) or RNFL thickness map (Fig 2B) varied according to where the optic disc center was located. The automatically calculated optic disc center was used as a reference point. We manipulated the optic disc center from 0 to 0.3 mm in 0.03-mm intervals in horizontal (temporal and nasal) and vertical (superior and inferior) directions and 0 to 0.42 mm in 0.042-mm intervals in diagonal (superotemporal, superonasal, inferonasal, and inferotemporal) directions. The 0.03-mm (equal to 1 pixel) or 0.042-mm (equal to 1 diagonal pixel) intervals were the minimum evaluable scale in Cirrus HD-OCT. Then, RNFL thickness data were resampled with the following: (1) a 3.46-mm diameter circle; and (2) between a 2.5-mm diameter circle and a 5.4-mm square with respect to the manipulated optic disc center location (Fig 2). Resampled data at a 3.46-mm diameter circle were reconstructed to average, temporal, superior, nasal, and inferior parameters. The Error (or difference) between original and resampled RNFL measurements was calculated. For statistical analysis, data of left eyes were converted into a right eye format. The computer program used for these analyses was written using MATLAB R2012a.

All subjects had various initial optic disc center positions (range: -0.3 to 0.3 mm in x and y axes) and disc area (range: 1.04 to 4.09 mm^2^). RNFL measurements were analyzed within a 5.4-mm square area due to the variety of initial optic disc center locations. In addition, the optic disc center manipulation of 0.3 mm required an extra 0.3-mm space in the same direction (Fig 2B). If there was insufficient extra space, resampling was done outside the scanning area where no available RNFL thickness data existed. Therefore, comparison of the RNFL thickness map with optic disc center displacement was available within a 4.8-mm square area in a 6-mm square scanning area. The optic disc area contained no RNFL components, so a central circular area 2.5 mm in diameter was excluded from analysis.

### Statistical Analyses

Statistical analyses were performed using the R statistical programming language (ver. 3.1.2; R Foundation, Vienna, Austria). Because each subject had repeated RNFL measurements for each optic disc center manipulation (10 scales in 8 directions), linear mixed-effects models were fitted to RNFL measurements. Repeated measures ANOVA with Tukey's multiple comparison was performed to assess for error between original and resampled circumpapillary RNFL measurements. P-values of 0.05 or less were considered statistically significant.

To determine the clinically tolerable error threshold of circumpapillary RNFL thickness according to optic disc center displacement, we considered the test-retest variability of Cirrus HD-OCT. Suh et al. [7] reported that test-retest standard deviation (TRT-SD) in normal eyes was 1.93 μm in average, 2.28 μm in temporal, 4.11 μm in superior, 3.21 μm in nasal, and 3.83 μm in inferior RNFL thickness. We calculated the tolerance limit using the formula 1.645 x √2 x TRT-SD [8] with the following results: 1) 4.49 μm (4.85%, divided by mean thickness) in average; 2) 5.30 μm (7.68%) in temporal; 3) 9.56 μm (8.11%) in superior; 4) 7.47 μm (11.39%) in nasal; and 5) 8.91 μm (7.41%) in inferior RNFL thickness. These results were strictly applied to our threshold criteria. In this study, 5% or less variability between original and resampled RNFL measurements was defined as tolerable error, 5 to 10% as cautious, and 10% or more as unreliable. When all circumpapillary RNFL parameters (average, temporal, superior, nasal, and inferior segments) satisfied the tolerable error criterion, we regarded optic disc center displacement as tolerable for clinical use. If one of RNFL parameters included cautious or unreliable error, we regard as cautious or unreliable for clinical use.

The tolerable threshold for the RNFL thickness map needed different criteria, because there were tens of thousands of RNFL measurements. When the area of 5% or less variability occupied more than 80% of the RNFL thickness map, we regarded this map as tolerable for clinical use. When the area of 10% or less variability occupied more than 80% of RNFL thickness map, we regarded as cautious for clinical use. The area of 10% or more variability was defined as unreliable zone. The results were plotted in the ‘displacement error-encoded map’ using MATLAB software.

## Results

Two hundred and thirty-nine healthy subjects were enrolled in this study. The serious errors of optic disc margin detection by OCT’s built-in algorithm were observed in 9 cases (3.8%). Seven (2.9%) were excluded due to an initial optic disc center position > 0.3 mm. Two hundred and twenty-three healthy subjects were included in final analysis (mean age, 55.4 ± 12.7 years; mean deviation, -0.29 ± 1.13 dB; Average RNFL thickness, 102.3 ± 6.6 μm). Subject demographics are summarized in Table 1.

**Table 1:** Characteristics of healthy subjects.

N	223
Age (yrs)	55.4±12.7
Sex (male:female)	116:107
Intraocular pressure (mmHg)	16.7±3.6
Signal strength	8.5±0.9
Refractive error (diopters)	-0.09±1.43
MD (dB)	-0.29±1.13
PSD (dB)	1.74±0.70
Disc area (mm^2^)	2.14±0.53
Rim area (mm^2^)	1.31±0.25
Average RNFL thickness (μm)	102.3±6.6

MD = mean deviation; PSD = pattern standard deviation; RNFL = retinal nerve fiber layer

**Fig 3:**
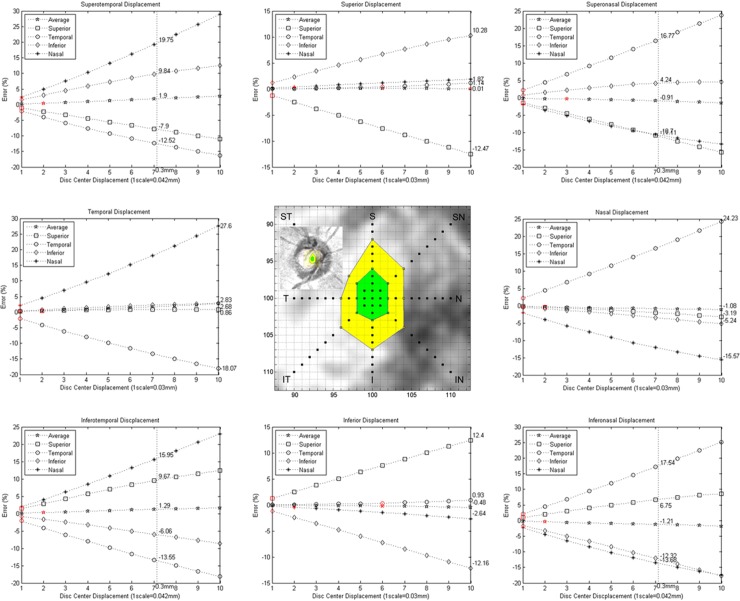
Optic disc center displacement and circumpapillary RNFL thickness error. Average RNFL thickness showed the smallest error for optic disc center displacement in 8 directions. The red marks on graphs represent the first significantly different optic disc center displacement between the original and resampled RNFL measurements on repeated measures ANOVA. The central green and yellow zone represents the maximum tolerable (5% or less variability) and cautious (10% or less variability) thresholds of optic disc center displacement. RNFL = retinal nerve fiber layer; ANOVA = analysis of variance

The effect of optic disc center displacement on circumpapillary RNFL thickness is described in Fig 3. Regardless of the direction of the optic disc center displacement, the average RNFL thickness had the least error between original and resampled RNFL measurements (range of error, -1.21 to 2.68%). Vertical displacement had a greater effect on superior and inferior RNFL thickness than temporal and nasal RNFL thickness. Horizontal and diagonal displacement had a greater effect on the temporal and nasal RNFL thickness than superior and inferior RNFL thickness. Measurements with significant difference between original and resampled RNFL by a repeated measure ANOVA test are indicated by red marks in Fig 3. Just 1 or 2 pixels displacement caused significant differences in nearly all circumpapillary RNFL parameters and displacement directions. The average and temporal RNFL thickness showed significant differences at ≥6 pixels displacement in the vertical direction. Considering RNFL measurement test-retest variability, the maximum tolerable thresholds (5% or less variability) of optic disc center displacement on circumpapillary RNFL measurement were distributed from 0.06 to 0.12 mm in 8 directions (Fig 3, green zone). The maximum cautious thresholds (10% or less variability) were distributed from 0.12 to 0.24 mm in 8 directions (Fig 3, yellow zone). These thresholds were more generous in vertical displacement than horizontal displacement.

**Fig 4:**
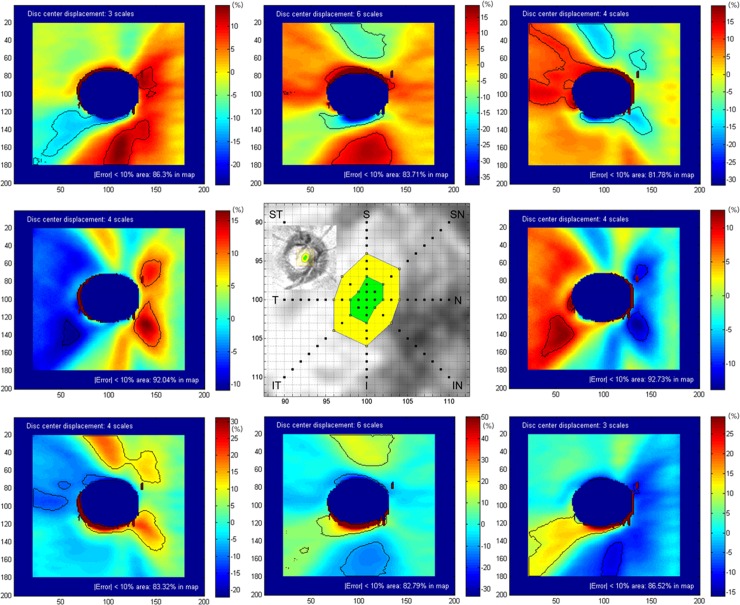
Optic disc center displacement and measurement error in the RNFL thickness map. Each displacement error-encoded map recorded differences between the original and resampled RNFL measurements at the maximum cautious threshold level (left upper corner). The black lines on the displacement error-encoded maps are the unreliable zones in which 10% or more variability was observed. The central green and yellow zone represents the maximum tolerable (5% or less variability occupying more than 80% of map) and cautious (10% or less variability occupying more than 80% of map) thresholds of optic disc center displacement. RNFL = retinal nerve fiber layer

The effect of optic disc center displacement on RNFL thickness map is displayed in the displacement error encoded map (Fig 4). Errors between original and resampled RNFL measurements were negatively increased in the region toward the direction of optic disc center displacement and positively increased in the opposite region. The unreliable zone (Fig 4, black lines on maps), in which 10% or more variability was observed, was located at the superior and inferior regions in vertical displacement (parallel distribution to the direction of displacement). In horizontal displacement, the unreliable zone was located at the inferotemporal, inferonasal, and superonasal regions (diagonal distribution to the direction of displacement). In diagonal displacement, the unreliable zone was frequently located at superotemporal or inferotemporal region (perpendicular distribution to the direction of displacement). Table 2 summarizes clinically important unreliable zones where is susceptible for glaucomatous damage. The maximum tolerable thresholds (≤5% variability occupying more than 80% of the RNFL map) of optic disc center displacement on the RNFL thickness map were distributed from 0.042 to 0.09 mm in 8 directions (Fig 4 center, green zone). The maximum cautious thresholds (≤10% variability occupying more than 80% of the map) were distributed from 0.12 to 0.18 mm in 8 directions (Fig 4 center, yellow zone). The thresholds were shaped as vertically elongated ellipses. Compared with the circumpapillary RNFL parameters, the tolerable or cautious thresholds ranges were narrower.

**Table 2:** Clinically important unreliable zone in terms of susceptible area for glaucomatous damage.

Direction	Unreliable zone	
	Decrease less than 10%	Increase more than 10%
Superotmporal (ST)	IT	I
Superior (S)	S	I
Superonasal (SN)	S	ST
Nasal (N)	-	IT
Inferonasal (IN)	I	IT
Inferior (I)	I	S
Inferotemporal (IT)	-	S
Temporal (T)	IT	-

**Fig 5:**
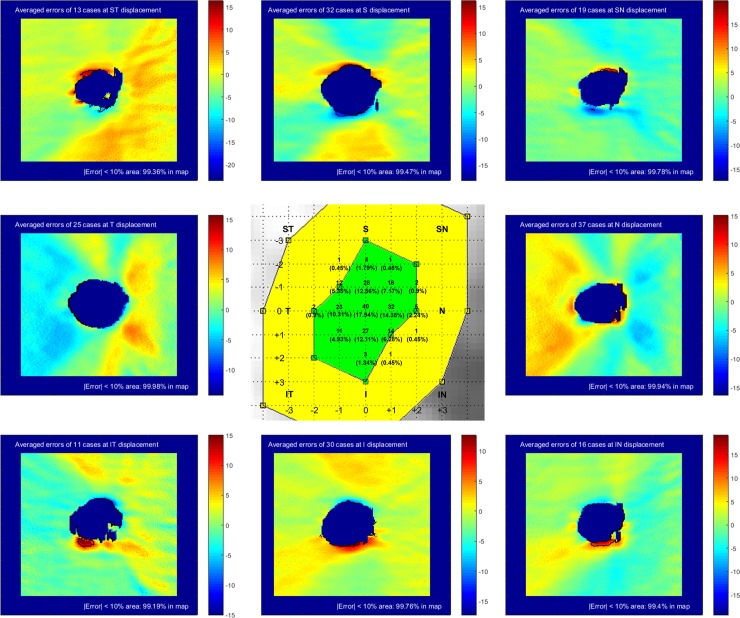
Optic disc center displacement between optic disc photograph and optical coherence tomography. The direction, magnitude, and frequency of optic disc center displacement were demonstrated with tolerable and cautious thresholds (center, green and yellow zone). The errors of RNFL measurements were displayed in the displacement error-encoded map at each direction. The black lines on the displacement error-encoded maps are the unreliable zones in which 10% or more variability was observed.

The errors of RNFL measurements and distribution of optic disc center displacement between optic disc photograph and OCT image were described in Fig 5. Of the 223 healthy eyes, 183 eyes (72.1%) had optic disc center displacement between optic disc photograph and OCT. The optic disc center of photograph was most frequently observed in nasal (37 eyes, 16.6%) direction based on the optic disc center of OCT. The optic disc center displacements occurred within -2 to +2 pixel units in x or y axis and satisfied the maximal tolerable threshold except 2 cases. The errors of RNFL measurements showed similar patterns with results from the above simulation. Almost all area (>99%) of the error encoded map did not exceed 10% and there was no clinically critical unreliable zone in error encoded maps.

## Discussion

The optic disc center is one of the most important landmarks in OCT RNFL imaging. Obtaining a well-centered scan is critical for reliable RNFL measurement [9]. In recent commercial SD-OCT, the ONH analysis algorithm automatically identifies the border of the optic disc and calculates the position of the optic disc center. An objective reference point can provide a consistent guideline for RNFL mapping as well as scan circle alignment. Consistent optic disc center identification is especially important in longitudinal observation, such as Guided Progression Analysis (GPA). ‘Translation of optic disc center’ and ‘rotation of OCT fundus’ are major strategies of GPA for registration of multiple images captured at different times. Although the ONH analysis algorithm provides reliable identification of the optic disc margin and center, the error of optic disc margin detection was reported to range from 0.5 to 17.6% in Cirrus HD-OCT [4–6]. In the present study, OCT’s built-in algorithm failed to detect optic disc margin in 9 cases (3.8%). Optic disc center displacement (or scan circle displacement) is possible in automated SD-OCT systems, and guidelines are needed to cope with optic disc center displacement in 200 x 200 RNFL thickness measurements.

In this study we evaluated 223 healthy eyes and found that the average RNFL thickness had the least variability in optic disc center displacement. The errors in average RNFL thickness at 0.3-mm displacement ranged from -1.21 to 2.68%. Average RNFL thickness was the only circumpapillary RNFL parameter that satisfied the test-retest variability tolerance limit in 8 directions of optic disc center displacement. Other sectoral RNFL parameters showed unreliable errors (-18.07 to 27.6%) exceeding the test-retest variability tolerance limit. These findings are consistent with previous studies using TD-OCT and SD-OCT. Vizzeri et al. [10] (a TD-OCT study of 16 healthy eyes) reported that average RNFL thickness was a robust parameter for scan circle displacement. Taibbi et al. [11] (a SD-OCT study of 18 healthy eyes) reported that changes exceeding the test-retest variability by scan circle displacement were minimally observed in average RNFL thickness. Because changes in sectoral RNFL would be compensated by corresponding changes in the opposite sector, average RNFL thickness could have minimal variation in optic disc center displacement.

A few studies have investigated the variability in circumpapillary RNFL thickness measurement as related to scan circle displacement. Vizzeri et al. [10] reported that scan circle shifts up to 300 μm may lie within the test-retest variability for average RNFL thickness measurements. Gabriele et al. [1] suggested wider threshold limits of over 600 μm. In our study, the maximum threshold for average RNFL thickness measurements was 240 μm. Although the RNFL thickness map is one of the main results of SD-OCT optic disc scan, RNFL thickness map variability resulting from optic disc center displacement has not been extensively studied. In this study, the maximum threshold of optic disc center displacement was up to 180 μm in the RNFL thickness map. Compared to circumpapillary RNFL parameters, RNFL thickness map had tighter threshold limits. Because optic disc center displacement affects 200 x 200 RNFL measurements, small changes lead to greater variability in the RNFL thickness map. Therefore, it requires greater attention to interpreting the optic disc center displacement in RNFL thickness map.

To the best of our knowledge, this is the first study demonstrating the effect of optic disc center displacement on RNFL thickness map. The displacement error encoded map (Fig 4) was the characteristic finding of this study. It demonstrated the distribution pattern of the unreliable zone. Even in cases where optic disc center displacement did not exceed the tolerable or cautious thresholds, areas with 10% or more variability were possible. Unfortunately, the unreliable zone was often located in regions critical for determining glaucomatous RNFL damage, such as the superotemporal, inferotemporal, superior, or inferior regions (Fig 4). Glaucomatous changes often start in the superior or inferior poles of the ONH [12]. In a recent study that analyzed RNFL thickness deviation maps, the RNFL defects were most frequently found at the inferotemporal (80.4%), followed by the superotemporal region (54.2%) [13]. Because optic disc center displacement could result in overestimation or underestimation of these regions susceptible to glaucomatous damage, the distribution pattern of unreliable zone should be considered when interpreting optic disc center displacement in RNFL thickness map.

The direction of optic disc displacement provides valuable information regarding where clinicians should focus their efforts (Table 2). Vertical displacement had the greatest effect on superior and inferior regions, while horizontal or diagonal displacement frequently led to the greatest changes in the superotemporal or inferotemporal regions. Moreover, the maximum tolerable or cautious thresholds of optic disc center displacement showed vertically elongated margins, meaning that the vertical displacement caused less error in RNFL thickness measurement than the horizontal displacement. Thus, the unreliable zone pattern and the threshold limit varied according to the direction of optic disc displacement. If optic disc center displacement is suspected, identifying the direction of displacement should be a top priority.

### How to deal with optic disc center displacement in clinical practice

Optic disc center displacement can be incurred by OCT built-in algorithm, because clinically visible optic disc margin is not always coincident with the termination of Bruch’s membrane. Reis et al. [14, 15] reported that there is an invisible extension of Bruch’s membrane internal to the clinically visible disc margin in most eyes. Although serious optic disc margin detection errors of OCT were excluded from this study, optic disc center displacement between optic disc photograph and OCT image were observed in 72.1%. Nevertheless, the errors of RNFL measurements did not exceed tolerable threshold and there was no clinically critical unreliable zone in error encoded maps. Our findings indicate that a subtle optic disc center displacement caused by usual regional variability of Bruch’s membrane opening and clinically visible disc margin frequently happens but more intensive adjustment is not necessarily beneficial.

**Fig 6:**
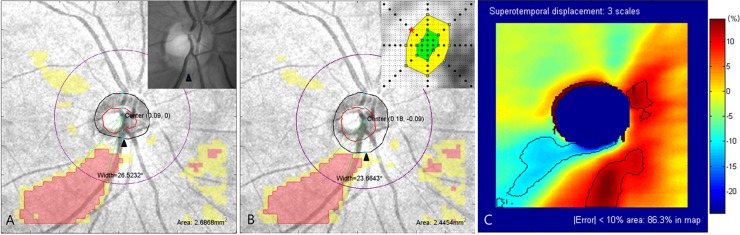
Adjustment of optic disc center displacement using Cirrus HD-OCT Research Browser. (A) The optic disc margin detection error (arrowheads) was highly suspect compared with the red-free photograph, and led to miscalculation of the optic disc center [0.09, 0 mm]. (B) Cirrus HD-OCT Research Browser provides an editing tool for adjusting the optic disc center and margin. After the optic disc center was adjusted to [0.18, -0.09 mm] by semi-automatic manipulation using the editing tool, the original shape of the optic disc margin was restored (arrowheads). Comparing two coordinates of the optic disc centers, superotemporal [-0.09, 0.09 mm] displacement (red asterisk) was observed. This corresponds to the boundary of the cautious threshold limit and should be interpreted with caution. (C) We can estimate or confirm the effect of optic disc center displacement using the displacement error-encoded map. In superotemporal displacement, the major clinical concern is the inferotemporal unreliable zone in which the greatest decrease is expected. Indeed, the red-coded area and its angular width increased 2.45 to 2.69 mm^2^ (9.8%) and 23.7 to 26.5° (11.8%). Minor changes in the superotemporal (increased yellow-coded area) and inferonasal region (decreased yellow- and red-coded area) were also consistent with the displacement error-encoded map. OCT = optical coherence tomography

In case of serious optic disc center displacement, Cirrus HD-OCT Research Browser supports an editing tool for adjusting the optic disc center and margin. The direction and magnitude of optic disc center displacement can be quantified using this tool. Fig 6 illustrates this process with a sample case. If clinicians encounter an obvious discrepancy of the optic disc margin between OCT and fundus photography (Fig 6A), correction of the optic disc center and margin would help to achieve reliable RNFL measurements (Fig 6B). Through semi-automatic manipulation of the optic disc center coordinates using the editing tool (Fig 6A, [0.09, 0 mm] before correction; Fig 6B, [0.18, -0.09 mm] after correction), appropriate optic disc margin could be restored (Fig 6A and 6B, arrowhead). In this case, the superotemporal [-0.09, 0.09 mm] displacement caused approximatively 10% changes in the area and angular width of RNFL defect. This study’s results (threshold limit and unreliable zone pattern) could contribute to the interpretation of revised RNFL measurement (Fig 6C). The displacement error encoded map was consistent with the actual changes in the above case.

**Fig 7:**
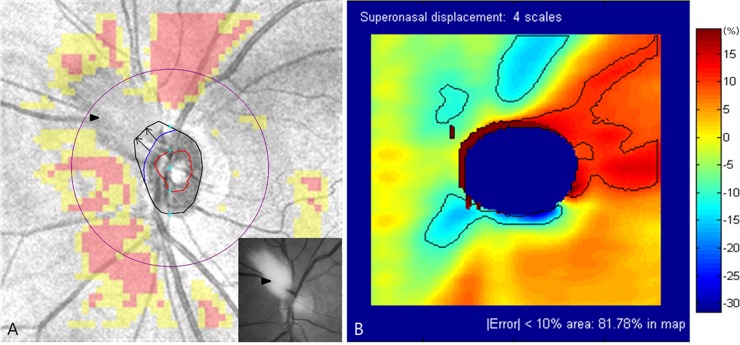
Interpretation of optic disc center displacement in case of myelinated retinal nerve fiber. (A) Myelinated retinal nerve fibers (arrowheads) interfered with the optic disc margin detection (black irregular circle) in the superonasal region. In cases with structural obstacles around the optic disc, the OCT algorithm usually failed to identify optic disc margin. Manual delineation (blue irregular arc) was performed by extending the visible surrounding optic disc margin. The direction of optic disc center displacement was estimated to be superonasal (arrows). (B) The unreliable zone should be interpreted with caution in the displacement error-encoded map. In superonasal displacement, there was a possibility of underestimation of RNFL thickness in superior, superonasal, and inferonasal regions. Part of the red- or yellow-coded areas in the RNFL deviation map might indicate false-positive RNFL thinning. In contrast, the superotemporal region was likely overestimated. Further investigation is needed for potential glaucomatous damage in this region. OCT = optical coherence tomography

**Fig 8:**
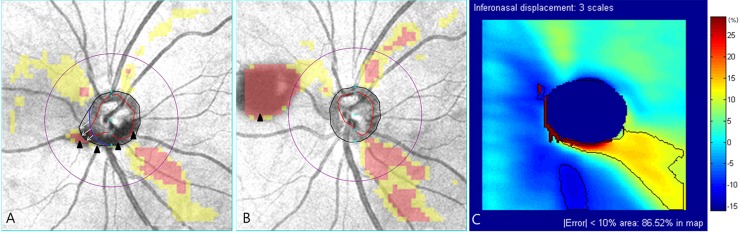
Interpretation of optic disc center displacement in case of floater. (A) Floater (arrowheads) interfered with the optic disc margin detection (black irregular circle) in the inferior region. Manual delineation (blue irregular arc) indicated that the direction of optic disc center displacement was estimated to be inferonasal (arrows). (B) At next follow-up exam (6 month interval), the floater (arrowhead) moved nasal side, and then optic disc margin was clearly detected. Although inferotemporal and superotemporal RNFL defects seemed to be progressed in the RNFL deviation map, there had been no evidence of such rapid progression in other structural and functional tests. Optic disc center displacement might affect the change of red and yellow-coded area. (C) In inferonasal displacement, there was greater chance of overestimation of RNFL thickness in inferotemporal region. Superotemporal region also was suspicious of mild overestimation. These were consistent with relatively small RNFL defects in first exam. RNFL = retinal nerve fiber layer

However, in cases with physical obstacles or structural abnormalities around the optic disc, the OCT algorithm usually fails to adjust the optic disc margin and center. In these cases, this study’s results could be a practical guideline for interpreting the effect of optic disc center displacement. Figs 7 and 8 demonstrates cases of myelinated retinal nerve fiber and floater interfering with optic disc margin detection. If Cirrus HD-OCT Research Browser is not available or fails to adjust the optic disc margin, we recommend the following steps: (1) determine the direction of optic disc center displacement (Fig 7A and Fig 8A), (2) select the displacement error-encoded map according to the direction in Fig 4, and (3) interpret RNFL measurements carefully considering the unreliable zone (Fig 7B and Fig 8C).

**Fig 9:**
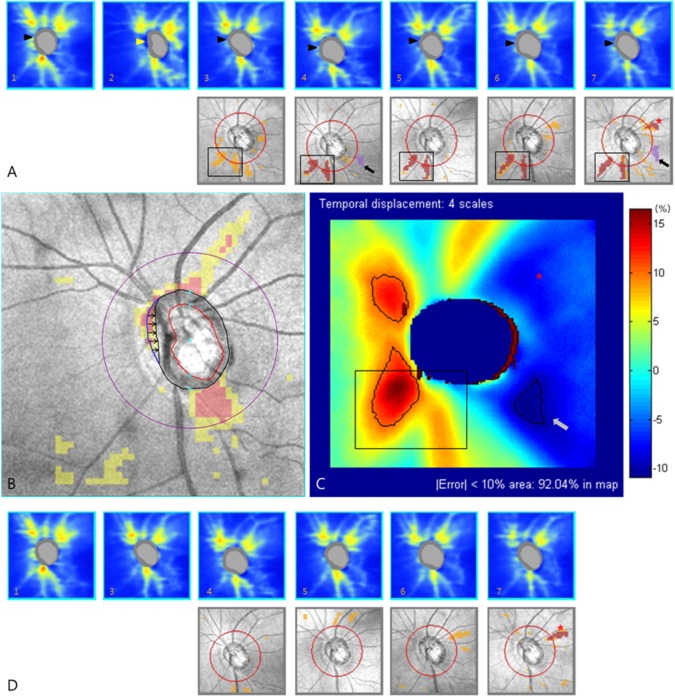
Interpretation of optic disc center displacement in Guided Progression Analysis (GPA). (A) At second baseline exam in GPA, the optic disc margin (yellow arrowhead) showed eccentric shape compared with other exams (black arrowheads). As a result, following RNFL thickness change maps showed RNFL progression at unusual region (inferonasal rectangles) and superotemporal region (asterisk). (B) Manual delineation (blue irregular arc) indicated that the direction of optic disc center displacement was estimated to be temporal (thin black arrows). (C) In temporal displacement, overestimated inferonasal region (rectangle) at baseline study might lead to false RNFL progression in GPA. In contrast, underestimated inferotemproal region (thick gray arrows) at baseline showed increasing RNFL thickness in follow-up exams (thick black arrows). Mild superotemporal underestimation (asterisk) at baseline might underestimate actual RNFL progression. (D) Using manual selection mode in GPA, we removed second exam from analysis. Abnormal RFNL progression at inferonasal region was proven to be the effect of optic disc center displacement. Superotemporal RNFL progression became larger and more obvious. RNFL = retinal nerve fiber layer

The optic disc center is especially important in GPA because it is a reference point for comparing several examinations. Optic disc center displacement at baseline exams may cause serial misguidance for RNFL progression in follow-up exams (Fig 9). Considering the effect of optic disc center displacement can improve the reliability of GPA.

This study examined only healthy eyes. Although the test-retest variability is independent of the severity of glaucomatous damage [16], it remains unclear whether optic disc center displacement variability in normal eyes applies to glaucomatous eyes; thus, further investigation is needed. Another limitation of this study is that the analyzed area is relatively small (4.8 x 4.8 mm^2^), and the effect of optic disc center displacement is not available at the peripheral region of the RNFL thickness map. Fortunately, recent studies reported that the RNFL defect and its progression were best detected at a distance of 2.14 and 2.00 mm from the optic disc center, respectively [17, 18].

In summary, optic disc center displacement had a considerable impact on whole RNFL thickness measurements. Understanding the effect of optic disc center displacement could contribute to the reliable RNFL measurements. Especially, it might improve the reliability of longitudinal OCT study in which accurate and consistent alignment is essential. The direction of optic disc center displacement correlated with a characteristic unreliable zone pattern and threshold limit. Identifying the direction of optic disc displacement is therefore highly recommended as the first step in managing the optic disc center displacement problem.
